# DISTRIBUTION AND DETERMINANTS OF LONG-ACTING INJECTABLE ANTIPSYCHOTIC MEDICATION USE AMONG OLDER ADULTS

**DOI:** 10.1093/geroni/igae098.0518

**Published:** 2024-12-31

**Authors:** Matthew Lohman, Mansi Verma, Paige Jones, Victoria Scott, Eve Fields

**Affiliations:** University of South Carolina, Columbia, South Carolina, United States; University of South Carolina, Columbia, South Carolina, United States; University of South Carolina, Columbia, South Carolina, United States; University of North Carolina Charlotte, Charlotte, North Carolina, United States; South Carolina Department of Mental Health, Greer, South Carolina, United States

## Abstract

While long-acting injectable antipsychotic medications (LAIs) are important components in the management of schizophrenia and related disorders, little is known about LAI use among older adults in real-world settings. We analyzed electronic health records from the South Carolina Department of Mental Health comprising 41,401 patients (n=8,481 aged > 60) who received ambulatory psychiatric services at one of 16 regional mental health centers in 2022. We compared use of eight types of first- and second-generation LAIs by demographic (age, race/ethnicity, gender, zip code, insurance source) and clinical characteristics (primary and secondary diagnoses, oral antipsychotic use). Logistic regression models were used to estimate associations between individual characteristics and likelihood of using LAI medications. In total, 1,400 adults age > 60 used LAIs in 2022, including 1,231 with schizophrenia or another psychotic disorder. Compared to younger adults, older adults were less likely to use LAIs but more likely to use first vs. second-generation LAIs (OR=2.19, p<.001). Controlling for diagnosis, insurance provider, and total services, older adults were significantly more likely to use LAIs if they were male (OR=1.40, p=.002), Black (OR=2.46, p<.001), Asian (OR=9.79, p=.001), and living in a suburban area (OR=1.33, p=.009). There were no significant demographic differences in oral antipsychotic use. Results suggest that LAI use among older adults differs substantially by sociodemographic characteristics while oral antipsychotic use does not. Understanding reasons for differences is important to determine and promote equitable access to and use of LAIs across racial/ethnic, geographic, and other sociodemographic groups of older adults.

